# Seasonal and Regional Variations in Nursing Workload in French Intensive Care Units: A National Study Using the Nursing Activities Score

**DOI:** 10.1155/jonm/6631036

**Published:** 2026-04-06

**Authors:** Laurent Poiroux, Pierre-Yves Blanchard, Anaëlle Caillet, Arnaud Bruyneel, Jérôme E. Dauvergne, the FNIR Study Group

**Affiliations:** ^1^ University of Angers-University of Rennes, Inserm, EHESP, Irset (Institut de Recherche en Santé, Environnement et Travail), UMR-S 1085 SFR ICAT, Angers, France, inserm.fr; ^2^ Department of Intensive Care Medicine, Tenon Hospital, Assistance Publique–Hôpitaux de Paris, Sorbonne University, Paris, France, sorbonne-universites.fr; ^3^ Department of Anesthesiology and Intensive Care, Lyon Sud University Hospital, Hospices Civils de Lyon, Pierre-Bénite, France, chu-lyon.fr; ^4^ Department of Health Economics, Hospital Management and Nursing Research, School of Public Health, Université Libre de Bruxelles, Brussels, Belgium, ulb.ac.be; ^5^ Department of Anaesthesiology and Critical Care, Nantes University Hospital, Laënnec Hospital, Nantes University, Nantes, France, chu-nantes.fr

**Keywords:** critical care nursing, ICU bed capacity, nursing staff, seasonal variation, workload

## Abstract

**Aim:**

To compare intensive care unit (ICU) nursing workload between spring–summer and winter and to examine factors associated with workload variation across French regions.

**Background:**

Critical care nursing workload is high and may fluctuate with seasonal demand and regional ICU bed capacity, challenging fixed‐staffing models.

**Methods:**

Secondary analysis of two nationwide prospective cross‐sectional surveys in French ICUs (April–July 2023; January–March 2024). Workload was measured using the Nursing Activities Score (NAS). Outcomes were NAS per patient, NAS per nurse, and NAS per nurse > 100%. ICU‐level linear mixed models assessed associations with season and regional ICU bed availability (below, versus, at, or above the national mean), adjusting for ICU characteristics.

**Results:**

Forty‐three ICUs contributed 2703 nurses and 18,772 patient NAS assessments (8759 NAS per‐nurse observations). At the individual level, median NAS per nurse was lower in spring−summer than in winter (124.3% vs. 127.1%; *p* value = 0.043), whereas NAS per patient was higher in spring−summer (61.3% vs. 59.4%; *p* < 0.001). The proportion of shifts with NAS per nurse > 100% was higher in winter (69.3% vs. 67.3%; *p* = 0.044), and the patient‐to‐nurse ratio was higher (2.06 ± 0.7 vs. 1.96 ± 0.7; *p* < 0.001). ICUs in regions with fewer ICU beds consistently showed higher NAS per nurse across seasons. In multivariable models, winter (β = 4.5, 95% CI: 2.3–6.7) and residence in under‐resourced regions (β = 19.2, 95% CI: 5.9–32.5) were associated with higher NAS per nurse.

**Conclusions:**

ICU nursing workload varies by season and regional ICU bed capacity; baseline workload frequently exceeds 100%, limiting surge adaptability and exposing limits of fixed‐staffing ratios.

**Implications for Nursing Management:**

Nurse managers may rely on objective and longitudinal workload information to support sustainable decision‐making in intensive care. Embedding indicators such as the NAS into staffing policies may facilitate anticipatory planning, reduce reactive staffing responses, and support patient safety and workforce stability.

**Summary**
• Reporting method
◦ This article follows the STROBE guidelines for the reporting of cross‐sectional studies
• Patient or public contribution
◦ No patient or public contribution.

## 1. Introduction

Intensive care unit (ICU) patients require a high level of nursing care [1], reflecting the intensity and complexity of interventions needed to ensure their safety and recovery. Numerous studies have reported the excessive workload experienced by critical care nurses [2–7]. These analyses consistently highlight the potential mismatch between the needs of critically ill patients and nurse staffing levels.

Given this excessive workload, any additional stressors, such as organizational strain or seasonal surges, may further destabilize this fragile balance. First, ICU strain has been shown to be a contributing factor to increased nursing workload [7]. This rise may be driven by increased patient admissions, shorter length of stay, and higher patient turnover [8]. It may also be linked to overtime work requested by managers during periods of elevated ICU admissions [9]. To address ICU organizational challenges during surge conditions, the Society of Critical Care Medicine recently issued a set of recommendations [10]. Determining team composition, staffing ratios, and the appropriate number of ICU beds is essential to maintaining care quality and system resilience. Due to the lack of a standardized definition of “ICU bed” and potential variations across healthcare systems, cross‐national comparisons remain challenging. Moreover, to our knowledge, no international recommendation has established an optimal number of adult ICU beds per capita. Available data estimated that for every 100,000 people, the United States has 31 ICU beds [11], Germany 24.6, and Canada 13.5 [12], well above the national ICU bed capacity in France, which stands at 6.35 per 100,000 adults [13, 14]. Although these national‐level data provide valuable insights, they reveal regional disparities [12, 13]. However, they also confirmed the necessity of further research to better understand how both shortages and surpluses of adult ICU beds affect patient outcomes, healthcare costs, or the workload of healthcare professionals.

Second, seasonal variations, understood as fluctuations in patient admissions and demand for nursing care, may further contribute to an imbalance between patients’ needs and care delivery capacity. Although seasonal variations in ICU nursing workload have not been directly studied, several indirect indicators suggest that workload pressure may fluctuate throughout the year, with potential consequences for both nurses and patients. For example, Bruyneel et al. [2], in a study assessing nursing workload in Belgian ICUs, reported an occupancy rate of 77.8% in winter versus 70.8% in spring. Similarly, in an emergency care setting, Zaboli et al. [15] identified marked seasonal fluctuations in triage nurses’ workload [15], with peaks in winter and summer, particularly in medium‐volume hospitals. This study also underscored the complexity of workforce management, emphasizing the need to ensure sufficient staffing and maintain adequate skill levels throughout the year.

In the ICU context, a better understanding of potential seasonal patterns could help nurse managers anticipate staffing needs and mitigate risks to the quality of care and nurses′ well‐being [16].

The aims of this study were to compare nurses’ workloads between spring−summer and winter and to analyze the potential factors influencing workload variations.

## 2. Methods

### 2.1. Study Design

This study represents a secondary analysis of data collected through two nationwide prospective cross‐sectional surveys previously conducted in French ICUs, each over a three‐month period. These studies correspond to the seasonal periods examined in the present analysis: Study 1 was conducted between April and July 2023 (spring−summer) and Study 2 between January and March 2024 (winter). Participation was voluntary in both studies. Throughout each study, nurses in the participating ICUs were asked to record their workload for each of the patients under their care over a two‐week period. Each ICU was free to choose the 2 weeks for workload data collection.

The results of this study are presented in accordance with the STROBE guidelines for observational studies [17].

### 2.2. Setting

Participation was open to all French ICUs, including intermediate care beds located within the ICU. At least 10 workload assessments per nurse for ICU patients had to be reported in each period for an ICU to be included in this analysis. Pediatric ICUs were excluded.

### 2.3. Data Collection

The data collected have been described elsewhere [3, 18]. In brief, these included organizational data relating to ICUs, such as shift organization (two or three shifts per 24 h), the number of beds, the type of ICU (medical, surgical, or general; intensive or intermediate care), the hospital’s type (academic or nonacademic, private), and the number of nurses per unit. The Nursing Activities Score (NAS) was collected by nurses at the end of their working hours and recorded on a secure digital platform, Sphinx, hosted by Nantes University Hospital.

To assess nursing workload, the NAS [19] was used, as it is considered the most appropriate tool for measuring workload [2]. Using 23 items (18 binary and 5 multiple‐choice) covering direct and indirect patient care, it provides a score ranging from 20% to 177%. It thus estimates 81% of nursing care provided in the ICU. It is also easy to read, as a score of 100% represents the allocation of one nurse per shift. Notably, it is also supported by a validated French translation [20]. Three outcomes were determined using the NAS: the NAS per patient per shift, the NAS per nurse, which represented the total NAS per patient assigned to one nurse per shift, and the prevalence of NAS per nurse exceeding 100%.

The territorial impact of nurses’ workload was studied by calculating the number of intensive care beds per 100,000 capita for each administrative region. This calculation incorporates the number of adult intensive care beds in metropolitan France (excluding intermediate care beds), as reported by the French Ministry of Health in 2023 [14], and the number of adult inhabitants in each region in 2022, based on the most recent census data [13]. This analysis focused exclusively on intensive care beds, as comprehensive and publicly accessible data concerning intermediate care beds were not clearly available. This variable was dichotomized by the national mean into a binary variable indicating whether a region was under‐resourced, defined as having a number of adult ICU beds per 100,000 inhabitants below this threshold (Supporting Table 1).

### 2.4. Statistical methods

The characteristics of the participating ICUs were described using either the mean and its standard deviation or the median and its interquartile range (p25%–p75%), depending on whether the data were normally distributed or not (graphically assessed) for continuous variables. Categorical variables were reported as numbers and percentages. Comparisons between periods were performed using Fisher’s exact test or chi^2^ as appropriate. Before conducting the analysis, outliers (values above 1.5 times the 75th percentile) were removed.

Both NAS per patient and NAS per nurse were analyzed from two complementary perspectives: at the individual level and at the ICU level. At the individual level, comparisons between the two study periods were made using the Wilcoxon test or Student’s *t*‐test as appropriate. On the other hand, an ICU‐level analysis was performed using a linear mixed model with NAS per patient or per nurse as the dependent variable. Thus, given the hierarchical structure of the data, with nurses nested within ICUs, this approach accounts for the intra‐ICU correlation while allowing estimation of an overall effect across ICUs. Random intercepts were specified for each ICU to capture unobserved ICU‐level heterogeneity. The study period was introduced as an independent variable, as well as descriptive variables: type of hospital, type of ICU, organization into two or three shifts per 24 h, and under‐resourced regions. Beta coefficients, representing a number of NAS points, were accompanied by their 95% confidence interval (95% CI). Each independent variable was individually tested in the mixed model. Only variables with *p* < 0.20 were retained in the final model [21]. The selection of the final model was guided by the lowest Akaike information criterion. We sought to identify interactions among variables and potential collinearity when the variance‐inflation factor exceeded 5. In the event of heteroscedasticity, a robust analysis of variance was performed. No imputation of the missing data was conducted. All analyses were two‐tailed, and statistical significance was set at *p* < 0.05. The statistical analysis was performed with R 4.4.1 (R, Vienna, Austria) [22].

### 2.5. Ethics

The two studies representing the spring−summer and winter periods of the present analysis were submitted separately to an ethics committee. A positive opinion was given on February 16, 2023, for the first study (number: 23‐28‐02‐161) and on September 15, 2023, for the second (number: 23‐106‐09‐150) by “Groupe Nantais d’Ethique dans le Domaine de la Santé “(GNEDS).

To comply with ethical standards and ensure the protection of personal information, each participating ICU and nurse was assigned a unique identification number to guarantee anonymity. All data and handling were in accordance with the applicable General Data Protection Regulations (EU 2016/679). No personal data from patients or nurses were gathered.

## 3. Results

**FIGURE 1:**
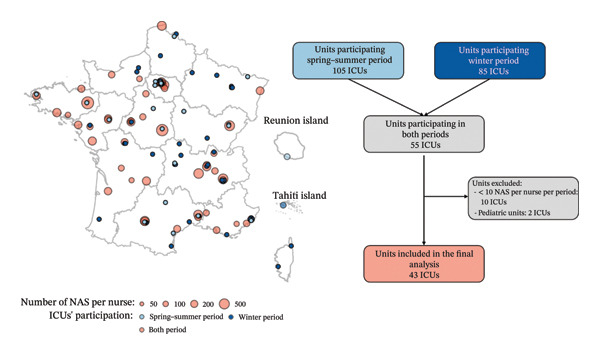
Flowchart of ICUs retained in the analysis.

The analysis encompassed 43 ICUs (Figure 1), and the main characteristics of the participating ICUs are displayed in Table 1. The cohort studied comprised 2703 nurses who collected 18,772 NAS per patient, 10,292 and 8480 during spring−summer and winter periods, respectively. The number of NAS per nurse analyzed was 8,759, with 4936 in the spring−summer period and 3823 in the winter period.

**TABLE 1:** Characteristics of the participating ICUs.

Variable	Overall (*n* = 43)
Regional ICU beds capacity	
Fewer resource ICUs	25 (58%)
Higher resource ICUs	18 (42%)
Hospital type	
Academic hospital, *n* (%)	26 (60.5)
Nonuniversity hospital, *n* (%)	15 (34.9)
Private hospital, *n* (%)	2 (4.6)
Type of ICU	
Medical, *n* (%)	16 (37.2)
General, *n* (%)	14 (32.6)
Surgical, *n* (%)	13 (30.2)
Number of beds	15 [12–22]
ICU beds	15 [11–20]
Shift organization	
2 shifts per 24 h, *n* (%)	39 (90.7)
3 shifts per 24 h, *n* (%)	4 (9.3)
Number of nurses per ICU	62 [50–80]
Number of NAS/nurse collected per ICU	200 [163–286]

*Note:* Legend: Variables are presented as a number (%) or median and its interquartile range (IQR).

Abbreviations: ICU, intensive care unit; NAS, Nursing Activities Score.

**FIGURE 3:**
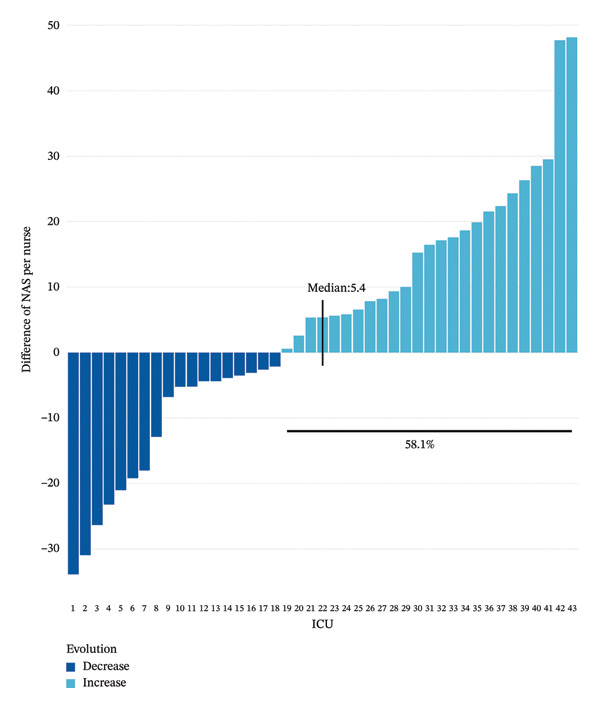
Evolution of NAS per nurse between the spring−summer and winter periods. Each bar represents the difference in NAS per nurse between the spring−summer and winter periods. When the score has decreased, the bar is dark blue, and when it has increased, it is light blue. The median increase in NAS per nurse was 5.4 [−4.8; +17.4]. 25 (58.1%) ICUs experienced an increase in NAS per nurse.

At the individual level, the median NAS per nurse was significantly lower in the spring−summer period than in the winter period, with 124.3% [86.5%–164.6%] vs. 127.1% [89.9%–166.2%] (*p* value = 0.043), respectively. The median NAS per patient was significantly higher in the spring−summer period than in the winter period, with 61.3% [48.2%–80.3%] vs. 59.4% [48.1%–77.2%] (*p* value < 0.001) (Figure 2). The prevalence of a NAS per nurse greater than 100% was less frequent in the spring−summer period than in the winter period, 67.3% vs 69.3%, *p* value = 0.044). The patient‐to‐nurse ratio was significantly higher during winter compared to the spring−summer period (1.96 ± 0.7 vs 2.06 ± 0.7, *p* value < 0.001). Between the two periods, 25 (58.1%) ICUs increased their NAS per nurse and 15 (34.9%) by at least 10 points. Among them, 15 (60%) were located in regions with fewer ICU bed resources. The median increase in NAS per nurse was 5.4% [−4.8%; 17.4%] (Figure 3).

FIGURE 2NAS per nurse and NAS per patient at the individual level. Box plots showing the median NAS per nurse (a) and median NAS per patient (b) with respect to study periods, the interquartile range, and the probability density of overall data distribution. Bar plots represents the prevalence of NAS per nurse > 100% for each period (c). ^∗^ *p* < 0.05; ^∗∗∗^ *p* < 0.001. NAS: Nursing Activities Score.

**Figure:**
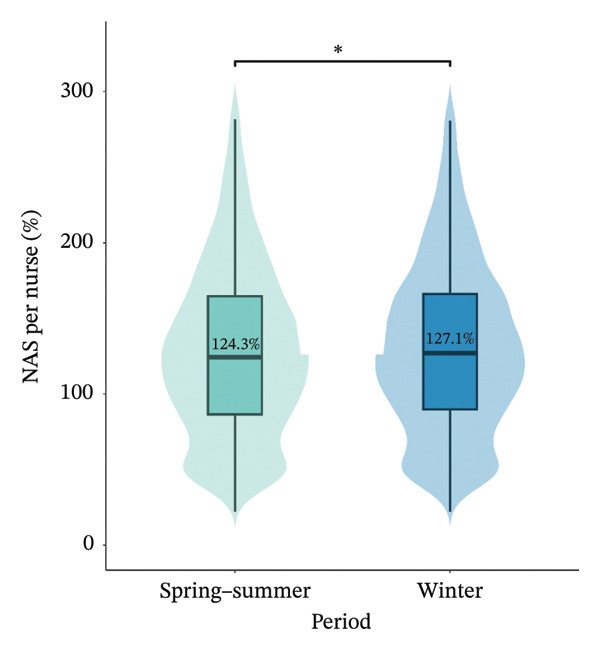
(a)

**Figure:**
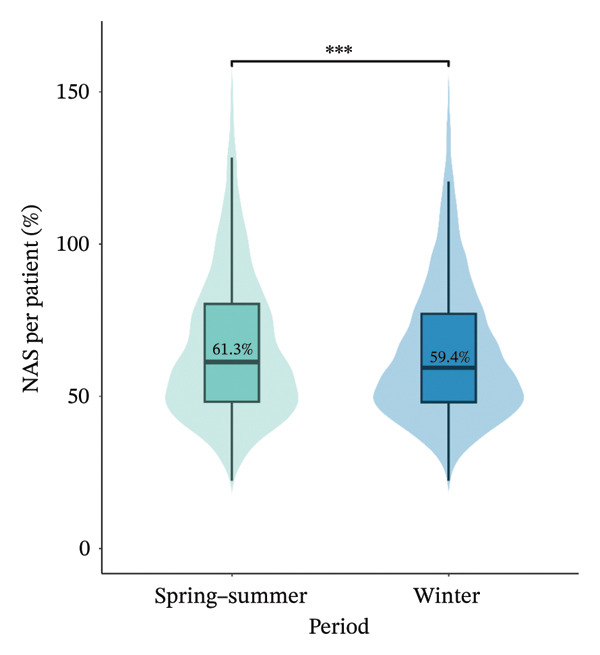
(b)

**Figure:**
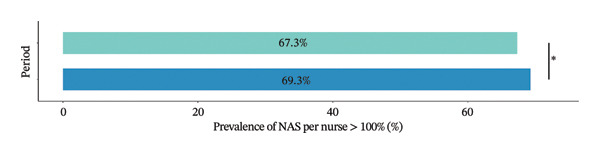
(c)

Comparisons of NAS items between the 2 periods are presented in Supporting Table 2. Items most significantly present during the winter period were those concerning respiratory support (7303 [71.0%] vs. 6280 [74,1%], *p* value < 0.001), care of artificial airways (4887 [47.5%] vs. 4357 [51.4%], *p* value < 0.001), and treatments for improving lung function (6327 [61.5%] vs. 5551 [65.5%], *p* value < 0.001).

ICUs located in regions with fewer ICU bed resources had a higher median NAS per nurse compared to ICUs from regions with higher ICU bed availability: 132.9% [98.4%–172.1%] vs. 105.9% [62.0%–146.0%] (*p* value < 0.001) during the spring−summer period and 137.8% [105.0%–173.2%] vs. 103.9% [59.2%–143.5%] (*p* value < 0.001) during the winter period (Supporting Table 3). A significant increase in NAS per nurse was observed in ICUs located in ICU‐bed‐under‐resourced regions (*p* value < 0.001), whereas no significant change was found in other ICUs (*p* value = 0.520).

**FIGURE 4:**
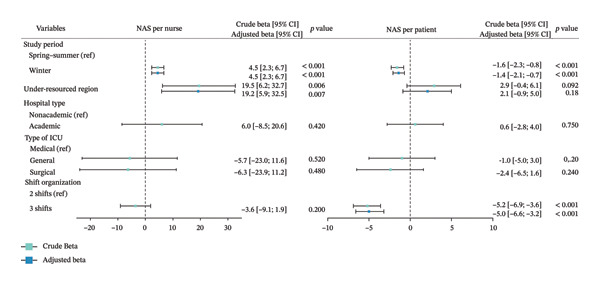
Association between NAS per nurse and NAS per patient with dependent variables. The results of the association between NAS per nurse and NAS per patient are expressed as NAS points with 95% confidence intervals (95% CIs). The forest plot shows the bivariate (crude beta) and multivariate linear mixed‐effects analyses (adjusted beta), with a random intercept specified for each ICU. Variables with a *p* < 0.20 in the bivariate analysis were retained in the multivariable model. ICU: intensive care unit; NAS: Nursing Activities Score.

Figure 4 presents the results of the ICU‐level analysis of NAS per nurse and NAS per patient. Variables associated with NAS per nurse in the multivariable analysis were the winter period, with a coefficient (beta) of 4.5 [95% CI: 2.3; 6.7] and residing in ICU‐bed‐under‐resourced regions, with a coefficient of 19.2 [95% CI: 5.9; 32.5]. Other variables, such as shift organization, hospital type, or ICU type, were not associated with NAS per nurse. The winter period (−1.4 [95% CI: −2.1; −0.7]) and the shift organization (−5.0 [95% CI: −6.6; −3.2]) were significantly associated with the NAS per patient according to the multivariable analysis, while being in ICU‐bed‐under‐resourced regions, the hospital type, and the type of ICU were not.

## 4. Discussion

This multicenter study highlights a significant seasonal increase in ICU nurse workload, as measured by the NAS, during winter compared to spring−summer. This increase was even more pronounced in regions with fewer ICU bed resources. While the NAS per patient was slightly lower during winter compared to the spring−summer period, the NAS per nurse increased, probably indicating that workload intensity shifted toward patient volume rather than individual care complexity, as nurses were simultaneously responsible for a higher number of patients. These findings have major implications for nursing management in critical care, particularly in workforce planning, team cohesion, and the fair allocation of human resources.

While month‐to‐month variability in NAS has been reported in longitudinal studies [23], our results confirm that ICU nurse workload fluctuates with seasonal patterns, challenging the adequacy of fixed‐staffing models. The traditional approach of maintaining a single staffing baseline throughout the year may overlook these variations and underestimate needs during periods of peak activity. More concerning, however, is that the NAS per nurse remained above 100% even in the lower‐activity period. This suggests that the baseline workload is often close to being unsustainable, thereby limiting the capacity to adapt during periods of increased demand.

Nursing care remains largely invisible within hospital reimbursement models, despite accounting for more than half of total ICU expenditures [24, 25]. As a result, staffing levels are often determined by budgetary limits rather than workload demands, disregarding seasonal and regional variations, despite evidence showing that lower ICU bed availability is linked to higher per‐patient day costs [26]. This structural invisibility perpetuates chronic understaffing and inequitable resource allocation. Embedding validated workload metrics, such as the NAS, into funding and staffing policies may represent a potentially valuable strategy to ensure workforce planning reflects both patient needs and organizational sustainability.

From a nursing management perspective, this highlights the need to develop targeted staffing management strategies to address seasonal workload fluctuations and variations in patient acuity more effectively. Li et al. [1] suggest considering more flexible and dynamic staffing models in light of NAS‐based workload patterns. Nevertheless, current evidence indicates that flexible deployment should complement, rather than substitute, adequate baseline staffing. Griffiths et al. [27] showed that, when combined with a low baseline roster, a high dependence on flexible staffing may result in unintended cost‐related consequences for the healthcare system. In the context of peaks in demand, staffing plans with higher baseline levels appear to be more cost‐effective than those relying on lower baseline staffing supplemented by flexible staff.

Beyond seasonal effects, workforce sustainability remains a major concern in intensive care nursing. Turnover intention among critical care nurses ranges from 23% in Europe [28] to 28% globally [29], confirming that this is a widespread concern across healthcare systems. To address this challenge, developing managerial strategies aimed at strengthening retention is crucial [28, 29]. These include ensuring an adequate patient‐to‐nurse ratio, offering meaningful forms of professional recognition, and providing support systems to mitigate moral distress. Moreover, although research on the effectiveness of debriefing remains limited [30], our results suggest that managers should implement routine team debriefings alongside systematic assessments of staff well‐being and turnover risk, using validated tools to measure burnout, moral distress, and intention to leave. Finally, fostering a psychologically safe work environment, characterized by authentic leadership, inclusive communication, and opportunities for team cohesion, emerges as a critical managerial priority to sustain engagement [31, 32].

Altogether, these interventions illustrate a shift from reactive to proactive workforce management, placing organizational health at the core of ICU staff retention strategies. This becomes particularly necessary during surge periods, when nursing teams are frequently asked to demonstrate flexibility by extending working hours or accepting increased patient loads [9].

Beyond the seasonal pattern, this study underscores a persistent and significant disparity in workload between regions. Nurses working in areas with lower ICU bed capacity consistently reported higher NAS scores, regardless of the season. This highlights the structural inequalities that disproportionately affect some teams, regardless of their internal organization or effectiveness. Such disparities cannot be resolved at the ICU level alone. While nurse managers in ICU‐bed‐under‐resourced regions may implement creative and adaptive solutions, they are constrained by systemic limitations. Equitable allocation of ICU beds and staffing resources across the national territory should be considered a political and policy priority. These findings echo recent calls from the United States for strategic health planning that ensures fair access to critical care for all patients and fair working conditions for all healthcare professionals [11].

In this sense, nurse managers play a critical role as data‐driven advocates. Systematic workload monitoring can help generate the evidence needed to support regional and national efforts to rebalance resources and design more sustainable staffing models.

The persistent overload observed in this study also highlights the relevance of the current staffing benchmarks. The median NAS per patient remained well above 50% in both periods, supporting the evolution of the French staffing standard from a 2.5:1 to a 2:1 open bed‐to‐nurse ratio in adult ICUs. However, this potentially reduced ratio may still represent a minimum requirement rather than an optimal threshold, particularly during the winter period, as seasonal variations are now known to influence nursing workload.

Staffing ratios serve as important administrative reference points and are intended as the minimum safety thresholds. However, they are neither evidence‐based nor designed to support real‐time workload monitoring or to align staffing levels with actual care needs. As previously demonstrated [33], adopting NAS in routine practice can support real‐time staffing decisions, longitudinal workload monitoring, and justification of staffing requests based on objective data for nurse managers. Therefore, this study supports the idea that staffing models may need to evolve beyond fixed ratios toward dynamic, data‐informed frameworks that better reflect clinical reality and promote both patient safety and staff sustainability.

## 5. Implication for Nursing Management

Our results highlight the dynamic nature of nursing workload in intensive care settings. Accordingly, managerial decision‐making in intensive care should be supported by objective and longitudinal information that allows variability in care demands to be taken into account. In this perspective, embedding objective indicators of nursing workload (such as the NAS) into staffing and funding policies may contribute to better aligning workforce planning with organizational sustainability.

By supporting long‐term rather than short‐term decision‐making, staffing approaches informed by longitudinal assessment of bed occupancy and regular workload monitoring may help limit reliance on reactive staffing responses, whose immediate benefits can be offset by longer‐term consequences for care quality and workforce stability. Access to objective workload data may also enable nurse managers to propose, discuss, and negotiate staffing arrangements and bed availability with decision‐makers in ways that are better aligned with care demands, patient safety, and organizational sustainability.

### 5.1. Strengths and Limitations

This study drew on a large national dataset, a validated workload metric, and the integration of both patient‐level and nurse‐level analyses. Its multicenter design enhances generalizability within the French ICU system. However, several limitations must be acknowledged. First, the NAS does not capture the emotional or ethical dimensions of workload, which are critical in ICU nursing. Second, our study did not include staff‐reported outcomes such as burnout, satisfaction, or perceived safety, which would complement the objective measures. Third, our study also excluded data from intermediate care units due to the wide variety of types of intermediate care units (hematology, neurovascular, cardiology, gastroenterology, etc.) and the great difficulty in distinguishing them from intermediate care related to intensive care based on publicly available data. Finally, although the French context offers robust data, comparisons with other countries require caution due to systemic differences.

## 6. Conclusion

This study confirms that ICU nursing workload varies significantly by season and region, with higher levels observed during winter and in ICU‐bed‐under‐resourced regions. These findings show the limitations of fixed‐staffing models and underscore the need for dynamic workforce planning strategies, emphasizing baseline staffing, team stability, and NAS tools for decision‐making. Proactive staffing policies and workload monitoring may enhance staff retention and continuity of care during surge periods. Regional workload disparities indicate the need for policy engagement to ensure equitable access to critical care. Overall, this study supports shifting from ratio‐based staffing to data‐informed frameworks that respond to patient acuity and systemic constraints, ensuring safe care and sustainable working conditions.

## Author Contributions

Laurent Poiroux: conceptualization, visualization, writing−review and editing, and supervision. Pierre‐Yves Blanchard: validation and writing−review and editing. Anaëlle Caillet: validation, reviewing, and editing. Arnaud Bruyneel: validation and writing−review and editing. Jérôme E. Dauvergne: conceptualization, software, formal analysis, data curation, visualization, and writing−review and editing.

## Funding

This research received no specific grant from any funding agency in the public, commercial, or not‐for‐profit sectors.

## Disclosure

All authors approved the final version of the report.

## Ethics Statement

The two studies representing the spring−summer and winter periods of the present study were submitted separately to an ethics committee. A positive opinion was given on February 16, 2023, for the first study (number: 23‐28‐02‐161) and on September 15, 2023, for the second (number: 23‐106‐09‐150) by “Groupe Nantais d’Ethique dans le Domaine de la Santé “(GNEDS). All data and its handling were in accordance with the applicable General Data Protection Regulations (EU 2016/679).

## Consent

The authors affirm that signed informed consent was obtained from all individual participants included in this study.

## Conflicts of Interest

The authors declare no conflicts of interest.

## Supporting Information

The following supporting material is available online and provides additional details supporting the findings of this study:

‐ Supporting Table 1: Number of ICU beds per 100,000 adults in each French metropolitan region.

‐ Supporting Table 2: NAS items by study period.

‐ Supporting Table 3: Comparison of NAS per nurse according to regional ICU bed allocation and study period.

## Supporting information


**Supporting Information** Additional supporting information can be found online in the Supporting Information section.

## Data Availability

The datasets used and analyzed in this manuscript are available from the corresponding author upon reasonable request.
