# Supplementary material for: Seasonal and Regional Variations in Nursing Workload in French Intensive Care Units: A National Study Using the Nursing Activities Score
Source: J Nurs Manag. 2026 Apr 6;2026:6631036. doi: 10.1155/jonm/6631036 (PMC13051825; doi:10.1155/jonm/6631036)
Supplement: Supplementary file 1 — Supporting Information Additional supporting information can be found online in the Supporting Information section. [file JONM-2026-6631036-s001.docx]

**Supplemental data related to the article entitled “Seasonal and regional variations in nurse workload in French intensive care units: a national study using the Nursing Activities Score”**

**Authors**

Laurent POIROUX^a^, Pierre-Yves BLANCHARD^b^, Anaëlle CAILLET^c^, Arnaud BRUYNEEL^d^, Jérôme E. DAUVERGNE^e^ for the FNIR Study group

^a^ Nursing Department, Health Faculty, Univ Angers, Univ Rennes, Inserm, EHESP, Irset (Institut de recherche en santé, environnement et travail) - UMR_S 1085, SFR ICAT, F-49000 Angers, France. [laurent.poiroux@univ-angers.fr](mailto:laurent.poiroux@univ-angers.fr).

^b^ Department of Intensive Care Medicine, Hôpital Tenon, Assistance Publique - Hôpitaux de Paris, Sorbonne Université, Paris, France. [pierre-yves.blanchard@aphp.fr](mailto:pierre-yves.blanchard@aphp.fr)

^c^ Department of Anesthesiology and Intensive Care, University Hospital Lyon Sud, Hospices Civils de Lyon, Pierre-Bénite, France. [anaelle.caillet@chu-lyon.fr](mailto:anaelle.caillet@chu-lyon.fr)

^d^ Health Economics, Hospital Management and Nursing Research Dept, School of Public Health, Université Libre de Bruxelles, Belgium. [Arnaud.bruyneel@ulb.be](mailto:Arnaud.bruyneel@ulb.be)

^e^ Nantes Université, CHU Nantes, Department of Anaesthesiology and Critical Care, Laënnec Hospital, F-44000 Nantes, France. [jerome.dauvergne@chu-nantes.fr](mailto:jerome.dauvergne@chu-nantes.fr)

**SUPPLEMENTAL DATA**

**Supplemental Table 1:** Number of ICU bed per 100,000 adults for each French metropolitan region

| **Region** | **ICU beds** | **Adult inhabitant** | **ICU bed per 100,000 adults** |
| --- | --- | --- | --- |
| Auvergne-Rhône-Alpes (ARA) | 524 | 8,163,884 | 6.42 |
| Bourgogne-Franche-Comté (BFC) | 199 | 2,803,977 | 7.10 |
| Bretagne (BR) | 177 | 3,422,845 | 5.17 |
| Centre-Val de Loire (CVL) | 173 | 2,581,597 | 6.70 |
| Corse (CO) | 24 | 351,276 | 6.83 |
| Grand Est (GE) | 384 | 5,560,079 | 6.91 |
| Hauts-de-France (HDF) | 440 | 5,998,916 | 7.33 |
| Île-de-France (IDF) | 782 | 12,380,964 | 6.32 |
| Normandie (NO) | 222 | 3,339,074 | 6.65 |
| Nouvelle-Aquitaine (NA) | 399 | 6,113,384 | 6.53 |
| Occitanie (OC) | 374 | 6,080,731 | 6.15 |
| Pays de la Loire (PDL) | 193 | 3,879,216 | 4.98 |
| Provence-Alpes-Côte d'Azur (PACA) | 291 | 5,170,312 | 5.63 |
| **Mean ICU bed per 100,000 adults** | **4,182** | **65,846,255** | **6.35** |


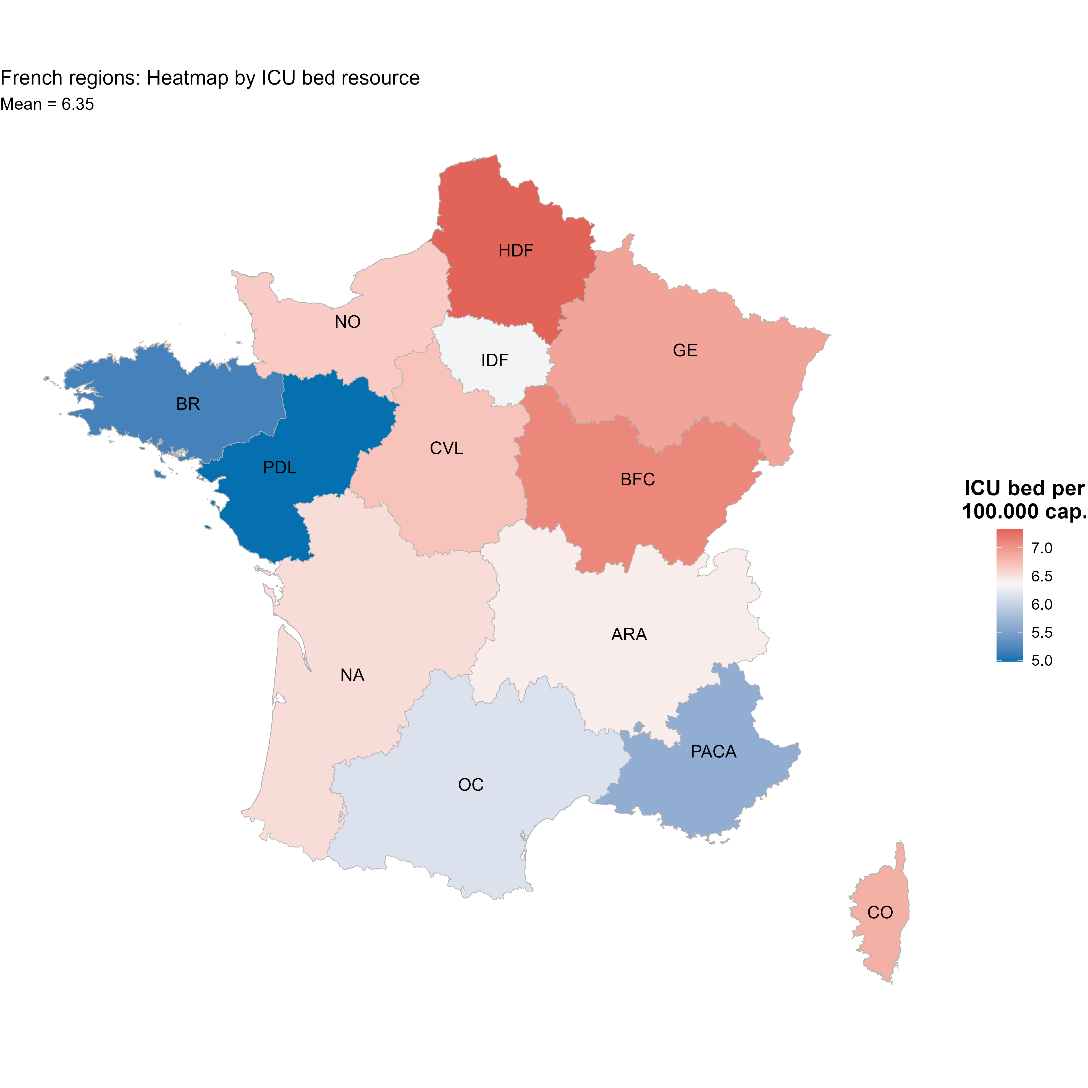


Legend: Public data on the number of intensive care beds and the number of adults living in each region of France. The map shows where these regions are located and identifies those with fewer ICU bed resources than others.

**Supplemental Table 2:** NAS items by study period

| NAS item | Spring – summer period  n = 10,292 | Winter  period  n = 8,840 | p |
| --- | --- | --- | --- |
| 1. Monitoring and titration  Normal  Higher than normal  Much higher than normal | 6,215 (60.4%)  3,270 (31.8%)  807 (7.8%) | 5,442 (64.2%)  2,491 (29.4%)  547 (6.5%) | **< 0.001**^a^ |
| 2. Laboratory, biochemical investigations | 8,182 (79.5%) | 6,803 (80.2%) | 0.221 |
| 3. Medication, vasoactive drugs excluded | 9,323 (90.6%) | 7657 (90.3%) | 0.501 |
| 4. Hygiene procedures  Normal  Higher than normal  Much higher than normal | 7,733 (75.1%)  2,191 (21.3%)  368 (3.6%) | 6,506 (76.7%)  1,772 (20.9%)  202 (2.4%) | **< 0.001** |
| 5. Care of drains, all (except gastric tube) | 4770 (46.3%) | 3,599 (42.4%) | **< 0.001** |
| 6. Mobilization and positioning, including procedures  up to 3/24 h  more than 3/24h or with 2 nurses  3 or more nurses | 5,187 (50.4%)  4,249 (41.3%)  856 (8.3%) | 4,472 (52.7%)  3,399 (40.1%)  609 (7.2%) | **< 0.001**^a^ |
| 7. Support and care of relatives and patient  Normal  Higher than normal | 8,586 (83.4%)  1,706 (16.6%) | 7227 (85.2%)  1,253 (14.8%) | **< 0.001** |
| 8. Administrative and managerial tasks  Courant  Higher than normal  Much higher than normal | 7,591 (73.8%)  2,397 (23.3%)  304 (3.0%) | 6,546 (77.2%)  1,786 (21.1%)  148 (1.7%) | **< 0.001** |
| 9. Respiratory support | 7303 (71.0%) | 6,280 (74.1%) | **< 0.001** |
| 10. Care of artificial airways | 4887 (47.5%) | 4,357 (51.4%) | **< 0.001** |
| 11. Treatment for improving lung function | 6,327 (61.5%) | 5,551 (65.5%) | **< 0.001** |
| 12. Vasoactive medication | 3,727 (36.2%) | 3,195 (37.7%) | **0.040** |
| 13. Intravenous replacement of large fluid losses | 2,627 (25.5%) | 2,085 (24.6%) | 0.141 |
| 14. Left atrium monitoring: pulmonary artery catheter | 3449 (33.5%) | 2,889 (34.2%) | 0.337 |
| 15. Cardiopulmonary resuscitation after arrest | 159 (1.5%) | 117 (1.4%) | 0.361 |
| 16. Hemofiltration techniques, dialysis techniques | 837 (8.1%) | 745 (8.8%) | 0.113 |
| 17. Quantitative urine output measurement | 8,157 (79.3%) | 6,801 (80.2%) | 0.113 |
| 18. Measurement of intracranial pressure | 569 (5.5%) | 358 (4.2%) | **< 0.001** |
| 19. Treatment of complicated metabolic acidosis/alkalosis | 1,020 (9.9%) | 696 (8.2%) | **< 0.001** |
| 20. Intravenous hyperalimentation | 1,803 (17.5%) | 1,546 (18.2%) | 0.206 |
| 21. Enteral feeding through a gastric tube | 4,097 (39.8%) | 3,670 (43.3%) | **< 0.001** |
| 22. Specific intervention(s) in the ICU | 1,900 (18.5%) | 1,392 (16.4%) | **< 0.001** |
| 23. Specific interventions outside the ICU | 1,002 (9.7%) | 696 (8.2%) | **< 0.001** |

Legend: All variables are expressed as numbers (%) for each modality. All tests were Fisher exact tests, except ^a^ Chi² test.

**Supplementary Table 3:** Comparison of NAS per nurse according to regional ICU bed allocation and study period

|  | **Spring-Summer period**  n = 5,786 | **Winter**  **period**  n = 4,470 | **p value** |
| --- | --- | --- | --- |
| Under-resourced regions | 132.9 [98.4 – 172.1] | 137.8 [105.0 – 173.2] | **< 0.001** |
| No under-resourced regions | 105.9 [62.0 – 146.0] | 103.9 [59.2 – 143.5] | 0.520 |
| ***p* value** | **< 0.001** | **< 0.001** |  |

Legend: Comparisons were performed with Wilcoxon test.

NAS: Nursing Activities Score
